# Involvement of collagen XVII in pluripotency gene expression and metabolic reprogramming of lung cancer stem cells

**DOI:** 10.1186/s12929-019-0593-y

**Published:** 2020-01-13

**Authors:** Han-Shui Hsu, Chen-Chi Liu, Jiun-Han Lin, Tien-Wei Hsu, Jyuan-Wei Hsu, Anna Fen-Yau Li, Shih-Chieh Hung

**Affiliations:** 1 Division of Thoracic Surgery, Department of Surgery, Taipei Veterans General Hospital, Taipei, Taiwan; 2 Institute of Emergency and Critical Care Medicine, School of Medicine, National Yang-Ming University, Taipei, Taiwan; 3 Division of Traumatology, Emergency Department, Taipei Veterans General Hospital, Taipei, Taiwan; 4 Faculty of Medicine, School of Medicine, National Yang-Ming University, Taipei, Taiwan; 5 Department of Pathology and Laboratory Medicine, Taipei Veterans General Hospital, Taipei, Taiwan; 6 Drug Development Center, Institute of New Drug Development, Institute of Biomedical Sciences, China Medical University, Taichung, 404 Taiwan; 7 Integrative Stem Cell Center, Department of Orthopaedics, China Medical University Hospital, Taichung, 404 Taiwan; 8 Institute of Biomedical Sciences, Academia Sinica, Taipei, 105 Taiwan

**Keywords:** Collagen XVII, Hexokinase 2, Lung cancer stem cells, Metabolic reprogramming, Oct4

## Abstract

**Background:**

Recent advancements in cancer biology field suggest that glucose metabolism is a potential target for cancer treatment. However, little if anything is known about the metabolic profile of cancer stem cells (CSCs) and the related underlying mechanisms.

**Methods:**

The metabolic phenotype in lung CSC was first investigated. The role of collagen XVII, a putative stem cell or CSC candidate marker, in regulating metabolic reprogramming in lung CSC was subsequently studied. Through screening the genes involved in glycolysis, we identified the downstream targets of collagen XVII that were involved in metabolic reprogramming of lung CSCs. Collagen XVII and its downstream targets were then used to predict the prognosis of lung cancer patients.

**Results:**

We showed that an aberrant upregulation of glycolysis and oxidative phosphorylation in lung CSCs is associated with the maintenance of CSC-like features, since blocking glycolysis and oxidative phosphorylation reduces sphere formation, chemoresistance, and tumorigenicity. We also showed that the Oct4-hexokinase 2 (HK2) pathway activated by collagen XVII-laminin-332 through FAK-PI3K/AKT-GSB3β/β-catenin activation induced the upregulation of glycolysis and maintenance of CSC-like features. Finally, we showed that collagen XVII, Oct4, and HK2 could be valuable markers to predict the prognosis of lung cancer patients.

**Conculsions:**

These data suggest the Oct4-HK2 pathway regulated by collagen XVII plays an important role in metabolic reprogramming and maintenance of CSC-like features in lung CSCs, which may aid in the development of new strategies in cancer treatment.

## Background

Cancer stem cells (CSCs), a subpopulation of cells within a tumor, were believed to confer tumorigenesis and resistance to standard treatment [1–3], and the identification of new CSC characteristics is important in developing new strategies for eradicating cancer. Recent advancement in tumor metabolism has led to the consideration that glucose metabolism could become a potential target for cancer treatment [4–6]. CSCs are supposed to get energy from different sources because of the complex spectrum of different microenvironments where they reside. Glioma stem cells (GSCs) consume less glucose and produce less lactate while maintaining higher ATP levels than their differentiated progeny [7]. Breast CSCs also produce less lactate, and have higher ATP content, maximum mitochondrial capacity and mitochondrial proton leak compared to their differentiated progeny [8]. These data suggest the energy of GSCs and breast CSCs rely on oxidative phosphorylation, which cannot be explained by the Warburg effect. In contrast, stem-like side population (SP) cells isolated from human non-small cell lung cancer (NSCLC) cells exhibit higher glycolytic activity compared to non-SP cells [9]. Therefore, metabolic features of different types of CSCs remain inconclusive.

Pluripotency genes, such as *Oct4* and *Nanog*, constitute the core regulatory network that suppresses differentiation-associated genes, thereby maintaining the pluripotency [10]. Oct4 has been known to regulate the transcription of the glycolytic enzymes hexokinase-2 (HK2) and pyruvate kinase muscle isozyme M2 (PKM2), which determine the glycolytic flux in embryonic stem cells [11]. The expression of *Oct4* has also been shown in human breast cancer stem-like cells, implicating its involvement in self-renewal and tumorigenesis via activating its downstream target genes [12]. However, the functional and mechanistic significance of these pluripotency genes in cancer is not well understood and little is known about the underlying mechanism controlling their expression.

Extracellular matrix (ECM) is essential for the uptake of extracellular nutrients and substrates for energy source. ECM also provides partitions that divid bulk cancer cells and CSCs, subsequently regulating the metabolic reprogramming of CSCs [13]. Collagen XVII, a type II integral transmembrane protein, is known as a structural component of hemidesmosomes that connects the intracellular space of epithelial cells to the underlying basement membrane. We have recently demonstrated that collagen XVII and laminin-332 were upregulated in CSCs in a variety of cancer types, including lung cancer, colorectal cancer, breast cancer, and brain cancer [14]. Collagen XVII and its downstream laminin-332 cooperate to form hemidesmosome-like structures, thereby mediating suspension survival in CSCs, and induce epithelial-mesenchymal transition (EMT) phenotypes through activation of the FAK-AKT-GSK3β pathways in lung CSCs [14, 15]. However, the roles of collagen XVII in pluripotency gene expression and metabolic reprogramming of lung CSCs have not been investigated. Here, we show that an aberrant upregulation of glycolysis and oxidative phosphorylation in lung CSCs is associated with the maintenance of CSC-like features, since blocking agents of glycolysis or oxidative phosphorylation reduce sphere formation, chemoresistance, and tumorigenicity. We then demonstrate that the Oct4-HK2 pathway, activated by collagen XVII-laminin-332 through activation of FAK-AKT/GSB3β/β-catenin, induces upregulation of glycolysis and maintenance of CSC-like features in lung CSCs. Finally, we show that collagen XVII, Oct4 and HK2 are valuable markers to predict the prognosis of patients with lung cancer.

## Materials and methods

### Cell line culture and reagents

The human NSCLC cell line A549 was obtained from the ATCC and grown in Ham’s F12 (Gibco, Grand Island, NY). Another human NSCLC cell line CL1–1 was kindly provided by Dr. Pan-Chyr Yang (Department of Internal Medicine, National Taiwan University Hospital, Taiwan, R.O.C.) and grown in Dulbecco modified Eagle medium (DMEM) (Gibco). HT29 (human colorectal cancer cell line) was obtained from the ATCC and grown in DMEM. Cells were cultured in F12 or DMEM containing 10 units/ml penicillin, 10 μg/ml streptomycin and 10% fetal bovine serum (FBS; Gibco). For enrichment of CSCs in spheroid culture, cancer cells were suspended in tumor sphere medium consisting of serum-free DMEM/F12, N2 supplement (Gibco), human recombinant epidermal growth factor (EGF) (20 ng/ml, PeproTech, Rocky Hill, NJ), and basic fibroblastic growth factor (bFGF) (10 ng/ml, PeproTech). Cell colonies > 50 μm in diameter and > 50% in area showing 3-dimensional structure and blurred cell margins were defined as spheres. Sphere numbers were counted at day 12 of culture. Treatment reagents included FAK inhibitor (10 μM) (Calbiochem, San Diego, CA), SB216763 (20 μM) (Sigma-Aldrich; St. Louis, MO), LY294002 (10 μM) (Abmole Bioscience, Houston, TX), and ICG001 (10 μM) (Abmole Bioscience).

### Western blot analysis

Cell extracts were prepared with M-PER (Pierce, Rockford, IL) plus protease inhibitor cocktail (Pierce) and protein concentrations are determined using the bicinchoninic acid (BCA) assay (Pierce). Aliquots of protein lysates are separated on SDS–10% polyacrylamide gels and transferred to PVDF membrane filters, followed by blocking with 5% blotting grade milk (Bio-Rad, Hercules, CA) in TBST (20 mM Tris–HCl pH 7.2, 137 mM NaCl, 1% Tween 20). Membranes are then probed with the indicated primary antibodies, reacted with corresponding secondary antibodies, and detected using a chemiluminescence assay (PerkinElmer Life and Analytical Sciences, Boston, MA). Membranes are exposed to X-ray film to visualize the bands (Amersham Pharmacia Biotech, Piscataway, NJ). Antibody against laminin-332γ2 (#sc-7652; 1:500) was purchased from Santa Cruz Biotechnology, Inc. (Santa Cruz, CA). Antibody against Sox2 (#GTX101507; 1:1000) was purchased from GeneTex (San Antonio, TX). Antibodies against collagen XVII (#ab28440; 1:1000) and Oct4 (#ab19857; 1:1000) were purchased from Abcam (Cambrige, MA). Antibodies against pAKT (Ser473, #3787; 1:1000), HK2 (#2867; 1:1000), PKM2 (#4053; 1:1000) and β-catenin (#8480; 1:1000) were purchased from Cell Signaling (Boston, MA). Antibodies against pFAK (#E011215; 1:1000) was purchased from EnoGene Biotech (New York, NY). Antibodies against pGSK3b (Ser9, #ADI-905-761–100; 1:1000) and Nanog (#ENZ-ABS270–0100; 1:1000) were purchased from Enzo Life Science, (Farmingdale, NY). Antibody against β-Actin (#A5441; 1:10000) was purchased from Sigma (St. Louis, MO).

### Extracellular flux analysis

Extracellular acidification rate (ECAR) and oxygen consumption rate (OCR) were measured using the Seahorse XF24 extracellular flux analyzer (Seahorse Bioscience, North Billerica, MA) according to the manufacturer’s instructions and as reviewed previously [16]. In brief, cells were plated in 24-well cell culture microplates (Seahorse Bioscience) at a density of 10^4^ cells per well to allow growth for 1 day and ensured 90% surface coverage. Media were replaced to XF base medium 1 h prior to analysis and incubated in a 37 °C incubator to stabilize the pH and temperature. In glycolysis analysis, different compounds were added in succession: glucose (10 mM), oligomycin (1 μM), and 2-deoxy-D-glucose (50 mM). These compounds are purchased as XF Glycolysis Stress Test kit. OCR were analysis from XF Mito Stress Test kit containing oligomycin (1 μM), FCCP (2 μM) and Rotenone/Antimycin A (0.5 μM). OCR and ECAR values were normalized to cell number.

### Glucose uptake and lactate assay

Glucose and lactate concentrations in the culture medium were determined by Glucose Uptake Colorimetric Kit and Lactate Colorimetric Assay Kit (Biovision, Milpitas, CA) according the manufacturer’s instructions using SpectraMax M5 multi- mode reader (Molecular Devices, Sunnyvale, CA).

### Mitochondrial activity assay

Mitochondrial activity was measured using Mitotracker dyes (Invitrogen, Carlsbad, CA). Cells were stained with a final concentration of 100 nM at 37 °C for 15 min, and then washed with PBS, fixed with 3.7% formaldehyde.

### Intracellular ATP analysis

Intracellular ATP levels were measured using ATP Colorimetric/Fluorometric Assay Kit (Biovision). 10^6^ cells were lysed in 100 μl ATP Assay Buffer and measured according the manufacturer’s instructions using SpectraMax M5 multi-mode reader (Molecular Devices).

### ALDH activity assay

ALDH enzymatic activities were analyzed using ALDEFLUOR assay (STEMCELL technologies, Vancouver, BC, Canada) according to the manufacturer’s instructions. In brief, cells were harvested, washed, and then resuspended to a concentration of 1 × 10^6^ cells /ml with the ALDEFLUOR™ assay buffer. Then 5 μl ALDH substrate was added into 1 ml cell suspension and mixed. As a negative control, 0.5 ml cell mixture was immediately transferred to control tube containing 5 μl specific ALDH inhibitor DEBA. After incubation at 37 °C for 30 min, cells were analyzed using a flow cytometer (FACS Canto II, BD Biosciences, San Jose, CA).

### Transfection of small interfering RNA

The small interfering RNAs (SiRNA) against Oct4 were purchased from Biotools (Taipei, Taiwan). Cells were transfected with Oct4-SiRNA using the TransIT-X2 transfection reagent (Mirus Bio LLC, Madison, WI) according to the manufacturer’s protocol.

### Lentiviral vector production and cell infection

The shRNA expression plasmids and bacterial clones for Col XVIIa1 (TRCN118937, TRCN118941) and HK2 (TRCN12545) were provided by the RNAi Core Facility, Academia Sinica (Taipei, Taiwan). Subconfluent tumor cells were infected with lentivirus in the presence of 8 μg/ml polybrene (Sigma-Aldrich). At 24 h post-infection, culture medium was replaced with fresh growth medium containing puromycin (4 μg/ml, Sigma-Aldrich) to select for infected cells after 48 h of infection.

### Cell transfection for col XVII overexpression

To establish human collagen XVII overexpression cell lines, cells were transfected with constructed plasmid pcDNA3.1 that had been inserted with the human collagen XVII gene (a gift from Doctor K. B. Yancey, University of Texas Southwestern Medical Center, Dallas, TX). Cells were transfected in serum-free OPTI-MEMI (Gibco) that contains plasmid DNA and using the TransIT-X2 transfection reagent (Mirus Bio LLC, Madison, WI). TransIT-X2 was mixed gently into the reaction solution, and the mixtures were incubated at room temperature for 15 min according to the manufacturer’s instructions. Cells were maintained in media with transfection complexes for 48 h. The cultures are then subjected to G418 (Enzo Life Science) selection.

### Growth and cytotoxicity analysis

Cells were plated at a density of 10^4^ cells/well in 96-well plates and incubated overnight. For growth assay, d-galactose or glucose (Sigma, St. Louis, MO) was added to make galactose or glucose media, respectively. Cells were then cultured in d-galactose (10 mM or 20 mM) without glucose, 2-deoxy-D-glucose (2-DG, 4 mM or 5 mM, Sigma) and metformin (2.5 mM or 5 mM, Selleckchem, Houston, TX) for 48 h, respectively. For cytotoxicity assay, cells were treated with cisplatin combined with 2-DG (4 mM) or metformin (2.5 mM) for 48 h. All assays were quantified using Cell Counting Kit-8 (Dojindo, Kumamoto, Japan).

### Tumor xenograft mouse model and treatment

Protocols involving mice were approved by the Institutional Animal Committee of Taipei Veterans General Hospital. NOD/SCID mice were purchased from the BioLASCO Experimental Animal Center (BioLASCO, Taiwan) and used for experiments at 6–8 weeks of age. Lung cancer cells were subcutaneously injected into mice with different doses and monitored for two months to evaluate tumor initiation. Absolute tumor-initiating frequency was calculated using the L-CalcTM Version 1.1 (Stem Cell Technologies, Vancouver, Canada). After sacrifice, tumors were resected and the expression of collagen XVII, Oct4 or HK2 was evaluated by immunohistochemistry (IHC). Anti-CK7 antibody (#M7018; 1:50, Agilent Dako, Santa Clara, CA), anti-collagen XVII antibody (#ab184996; 1:200, Abcam), anti-Oct4 polyclonal antibody (#2750; 1:50, Cell Signaling), anti-HK2 polyclonal antibody (#2867; 1:200, Cell Signaling) were used as the IHC primary antibodies.

For evaluation of lung metastasis ability, 3 × 10^5^ tumor cells were injected into the tail vein and lung tissue was harvested 12 weeks later. For tumor growth inhibition, 10^6^ tumor cells were subcutaneously injected into mice. Tumor size was measured and tumor volume was calculated as: (tumor volume = length x width^2^/2). Mice with tumors were randomly grouped when its size approached 50 mm^3^. For 2-DG treatment, mice were intraperitoneally injected with saline or 2-DG (800 mg/kg in saline, daily for 15 days). For metformin treatment, mice received metformin in drinking water (1 mg/ml) for 15 days. Tumor volume was measured every 3–4 days and tumor weight was determined after sacrifice.

### Quantitative real-time PCR (quantitative RT-PCR)

The total RNA was reversed transcribed in 20 μl using oligo dT and Superscript II RT (Invitrogen). The quantitative real-time PCR was performed using FastStart SYBR Green Master (Roche Applied Science, Mannheim, Germany) and ABI StepOnePlus Real-Time PCR System machine. The sequences of primer sets are listed in Additional file 9: Table S1.

### PCR array and data analysis

Total RNA was extracted using Trizol reagent (Invitrogen) and was analyzed by means of a Human Glucose Metabolism RT^2^ Profiler™ PCR Array (QIAGEN, Valencia, CA) according to the manufacturer’s instructions.

### Patients and IHC analysis of tumor specimens

Seventy-nine patients who underwent surgical resection for NSCLC between 2010 and 2015 were enrolled in this study. None of these patients received neoadjuvant chemotherapy or radiotherapy. The clinical data were collected by chart review. Paraffin blocks of tumor specimens were cut into 4-μm slices and then processed using standard deparaffinization and rehydration techniques, and then subjected to hematoxylin and eosin (HE) staining and IHC analysis. Anti-CK7 antibody (#M7018; 1:50, Agilent Dako), anti-collagen XVII antibody (#ab184996; 1:200, Abcam), anti-Oct4 polyclonal antibody (#ab19857; 1:200Abcam) anti-HK2 polyclonal antibody (#2867; 1:200, Cell Signaling) were used as the IHC primary antibodies. We used the score of positive tumor cell frequency and reaction intensity together to determine the result of IHC analysis. Score of positive tumor cell frequency: 0, < 25%; 1, 25~50%; 2, 50~75%; 3, > 75%. Score of reaction intensity: 0, no reaction; 1, weak; 2, moderate; 3, strong. Total score equaled the score of positive tumor cell frequency added the score of reaction intensity. If total score was more than two, we determined the expression of IHC was positive. On the contrary, if the total score was one or zero, the expression of IHC was negative.

### Statistical analysis

Values were shown as the mean ± standard deviation of the mean of measurements of at least three independently performed experiments. Student’s t-test or one-way ANOVA was employed. The correlations between IHC results and clinic pathological variables were analyzed by Pearson’s Chi-square test. Survival curves were calculated using the Kaplan-Meier method and comparisons were performed using the log-rank test. *P* < 0.05 was considered to be statistically significant.

## Results

### Lung CSCs increase glycolysis and oxidative phosphorylation

**Fig. 1:**
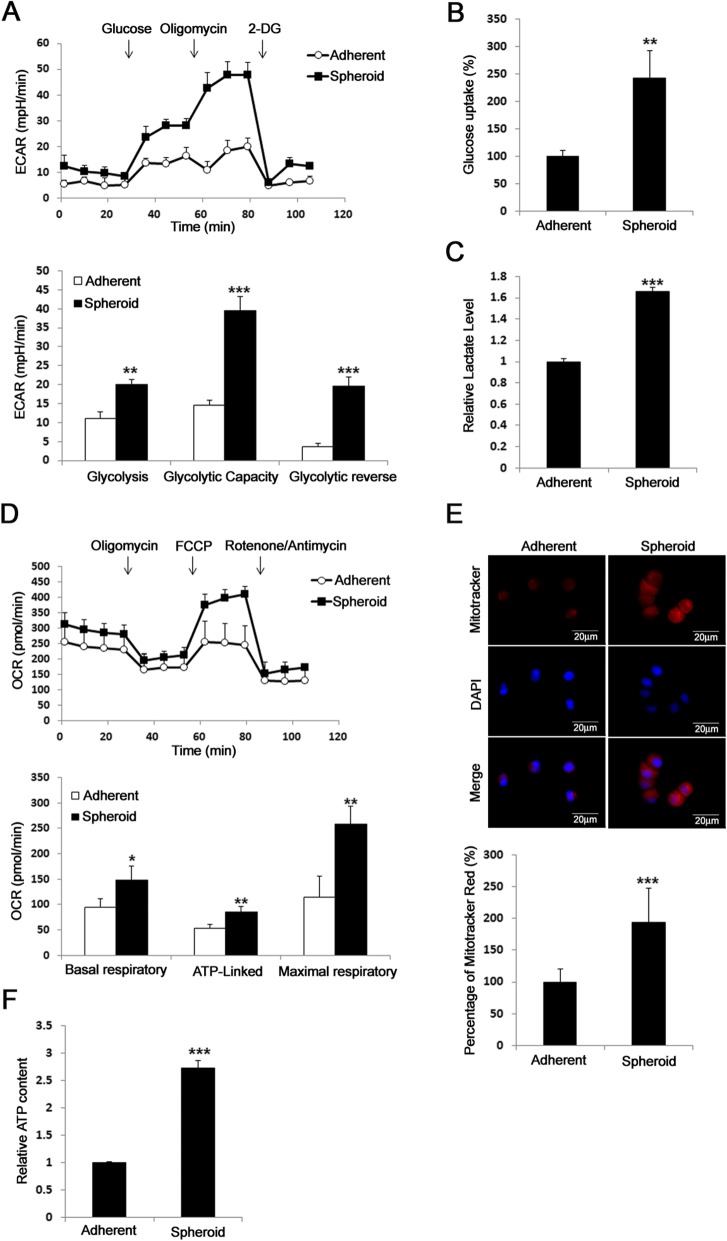
CSCs of lung cancer increase in glycolysis and oxidative phosphorylation. **a,** Lung cancer cells A549 cultured in spheroid medium showed increased extracellular acidification rate (ECAR) compared to cells without collagen XVII overexpression. **b** and **c,** The glucose uptake and lactate assay showed increased glucose consumption and lactate production in cells cultured with spheroid medium. **d,** The results of seahorse assay also showed increased OCR in cells cultured in spheroid medium. **e,** The mitotracker red staining showed increased mitochondria mass in cells cultured in spheroid medium. **f,** In ATP content assay, the data showed increased ATP production in cells cultured in spheroid medium

CSCs could be enriched by culturing primary or developed cancer cell lines in medium with serum reduction and the presence of growth factors such as EGF and FGF2 [17–19], in which cancer cells form spheres and become more capable of tumorigeneicity and resistant to chemotherapy. To demonstrate metabolic alteration in lung CSCs, we first showed that A549 in spheroid culture exhibited increased extracellular acidification rate (ECAR) (Fig. 1a), glucose uptake (Fig. 1b), and lactate production (Fig. 1c). Interestingly, A549 in spheroid culture also increased the oxygen consumption rate (OCR) (Fig. 1d), mitochondrial mass (Fig. 1e), and ATP production (Fig. 1f) compared to adherent culture. These data suggest that A549 lung CSCs have aberrant metabolic features.

### Involvement of collagen XVII in inducing CSC-like features in lung cancer cells

We have previously demonstrated the important roles of collagen XVII in suspension survival and the maintenance of EMT phenotypes in CSCs [14, 15]. Besides, collagen XVII has also recently been recognized as a master molecule and marker in hair follicle stem cells and orchestrates skin homeostasis and ageing [20, 21]. These data suggest that collagen XVII may have pleiotropic roles in regulating CSC phenotypes that may include tumorigenesis, metabolic reprogramming, and other CSC-like features. To evaluate the roles of collagen XVII in the maintenance of CSC-like properties, we first developed A549 lung adenocarcinoma cell lines with collagen XVII overexpression and silencing independently. As demonstrated before [14, 15], A549 in spheroid culture showed increased CSC-like properties, including sphere formation, upregulated ALDH activity, expression of pluripotency markers, tumorigenic potential, metastasis, and chemoresistance abilities when compared to A549 in adherent culture (Additional file 1: Figure S1). In comparison with the control transfections, collagen XVII overexpression in adherent culture increased sphere numbers, while collagen XVII silencing reduced sphere numbers in spheroid culture (Additional file 1: Figure S1A). Collagen XVII overexpression in adherent culture also increased ALDH activity (Additional file 1: Figure S1B), the expression of pluripotency markers (Additional file 1: Figure S1C), tumorigenic potential (Additional file 1: Figure S1D), metastasis ability when infused via tail vein (Additional file 1: Figure S1E), and chemoresistance abilities when treated with cisplatin (Additional file 1: Figure S1F). These data suggest that collagen XVII plays an essential role in inducing CSC-like features in lung cancer cells.

### Involvement of collagen XVII in metabolic reprogramming of lung CSCs

**Fig. 2:**
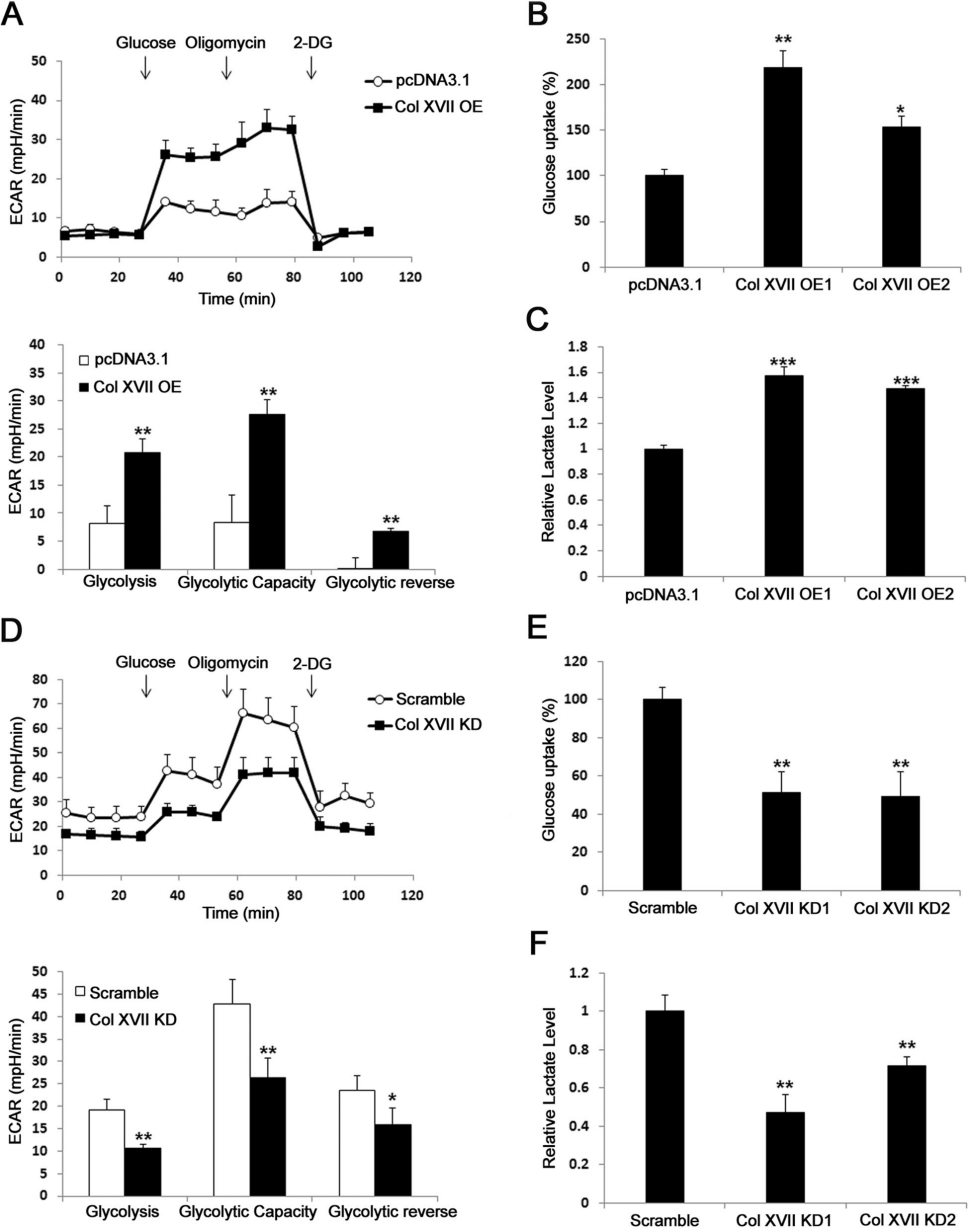
Collagen XVII is essential for increased glycolysis of lung cancer cells. **a,** Lung cancer cells A549 with collagen XVII overexpression (Col XVII OE) showed increased ECAR compared to parental cells. **b** and **c,** The glucose uptake and lactate assay showed increased glucose consumption and lactate production in cells with collagen XVII overexpression. **d,** Decreased ECAR was observed in lung cancer with collagen XVII knockdown (Col XVII KD). **e** and **f,** The glucose uptake and lactate assay showed decreased glucose consumption and lactate production in cells with collagen XVII knockdown

To demonstrate that collagen XVII plays an important role in metabolic reprogramming of lung CSCs, we first showed that collagen XVII overexpression in adherent culture increased ECAR (Fig. 2a), glucose uptake (Fig. 2b), and lactate production (Fig. 2c) compared to cells transfected with control plasmids. Moreover, collagen XVII silencing reduced ECAR (Fig. 2d), glucose uptake (Fig. 2e), and lactate production (Fig. 2f) in spheroid culture. Collagen XVII overexpression in adherent culture increased OCR (Additional file 2: Figure S2A), ATP production (Additional file 2: Figure S2B), and mitochondrial mass (Additional file 2: Figure S2C) compared to cells transfected with control plasmids. Moreover, collagen XVII silencing reduced OCR (Additional file 2: Figure S2D), ATP production (Additional file 2: Figure S2E), and mitochondrial mass (Additional file 2: Figure S2F) in spheroid culture. These data together suggest the essential role of collagen XVII in metabolic reprogramming of lung CSCs.

### Metabolic reprogramming is required for CSC survival and maintenance of CSC-like features

**Fig. 3:**
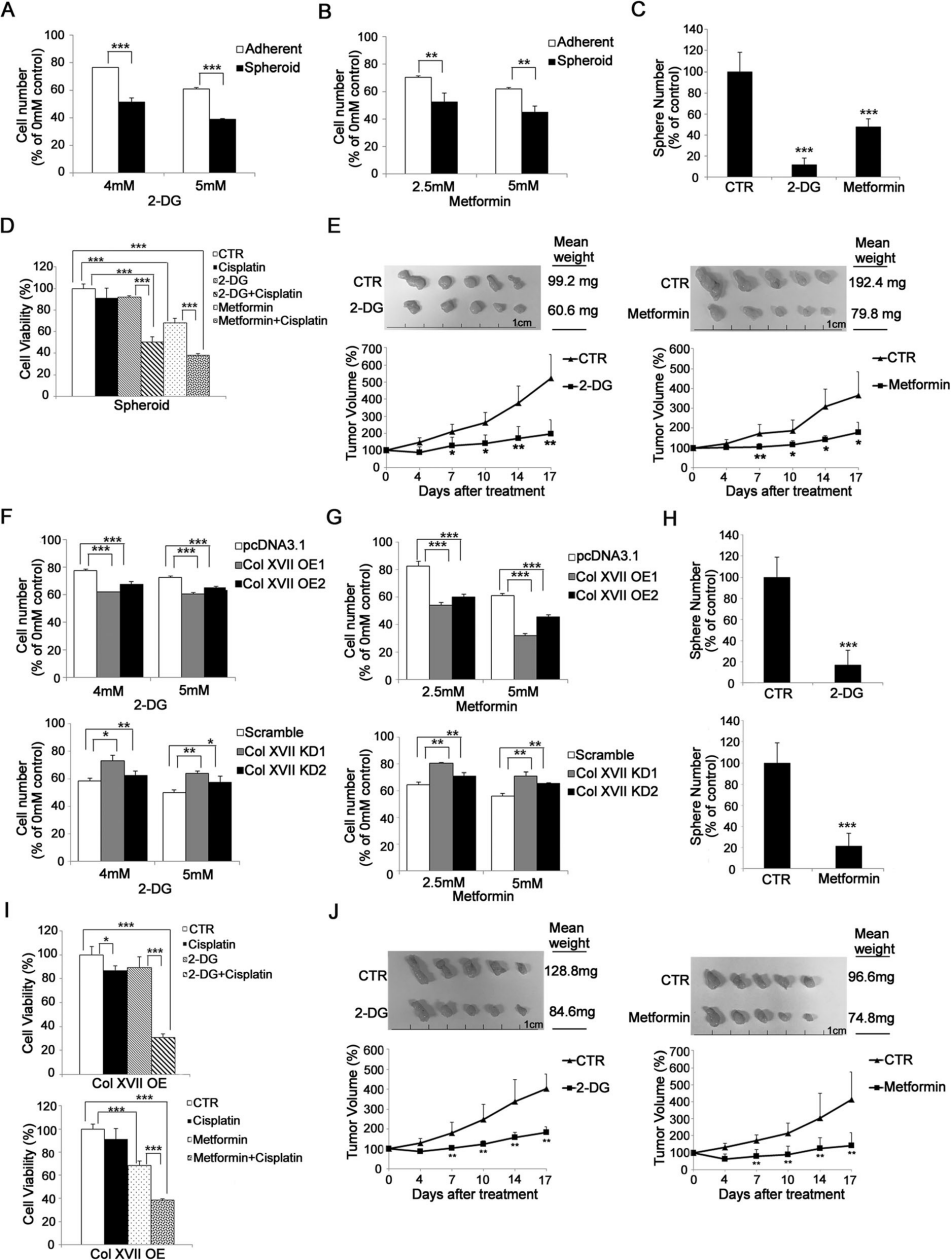
Lung cancer cells with overexpression of collagen XVII are resistant to the treatment of 2-DG and metformin in vitro and in vivo. **a** and **b,** When treated with 2-DG or metformin, lung cancer cells cultured with spheroid medium are more sensitive to the controls. **c,** Decreased sphere formation was noted when lung cancer cells cultured in spheroid medium were treated with 2-DG or metformin. **d,** When combined with cisplatin treatment, lung cancer cells cultured in spheroid culture were more sensitive to 2-DG or metformin treatment. **e,** In in vivo study, tumor size was significantly reduced when the mice were treated with 2-DG or metformin. **f** and **g,** Lung cancer cells with collagen XVII overexpression in monolayer culture were more sensitive to 2-DG or metformin. In contrast, cells with collagen XVII knockdown in spheroid culture are more resistant to 2-DG or metformin. **h,** Decreased sphere formation was noted when lung cancer cells with overexpression of collagen XVII were treated with 2-DG or metformin. **i,** Lung cancer cells with collagen XVII overexpression were more sensitive when treated with 2-DG or metformin in addition to cisplatin. **j,** In animal study, lung cancer cells with collagen XVII overexpression were injected s.c. in the back of the mice and treated with 2-DG or metformin. After 17 days, tumor size and weight were significantly decreased in the group of lung cancer cells with collagen XVII overexpression, compared to the control (CTR)

To demonstrate the requirement of metabolic reprogramming in CSC survival and the maintenance of CSC-like features in lung cancer, we first showed that A549 cells in spheroid culture were more sensitive to 2-deoxy-D-glucose (2-DG) (Fig. 3a), galactose (inhibitors of glycolysis) (Additional file 3: Figure S3A), and metformin (an inhibitor of oxidative phosphorylation)-induced cell death (Fig. 3b), compared to cells in adherent culture. Moreover, spheroid culture in the presence of 2-DG or metformin resulted in decreased sphere-forming abilities (Fig. 3c) and inhibited chemotoxic agent resistance (Fig. 3d), and formed smaller tumors in mouse xenograft models (Fig. 3e) in comparison with that in the absence of 2-DG or metformin. Collagen XVII overexpression also sensitized adherent cell culture to 2-DG (Fig. 3f), galactose (Additional file 3: Figure S3B), and metformin (Fig. 3g)-induced cell death. In contrast, collagen XVII silencing de-sensitized spheroid culture to 2-DG (Fig. 3f), galactose (Additional file 3: Figure S3C), and metformin (Fig. 3g)-induced cell death. Moreover, adherent culture with collagen XVII overexpression in the presence of 2-DG or metformin resulted in decreased sphere-forming abilities (Fig. 3h) and inhibited chemotoxic agent resistance (Fig. 3i), and formed smaller tumors in mouse xenograft models (Fig. 3j) in comparison with that in the absence of 2-DG or metformin. These data suggest that metabolic reprogramming is required for CSC survival and the maintenance of CSC-like features.

### HK2 is required for collagen XVII-mediated increase in glycolysis and CSC-like features in lung cancer

**Fig. 4:**
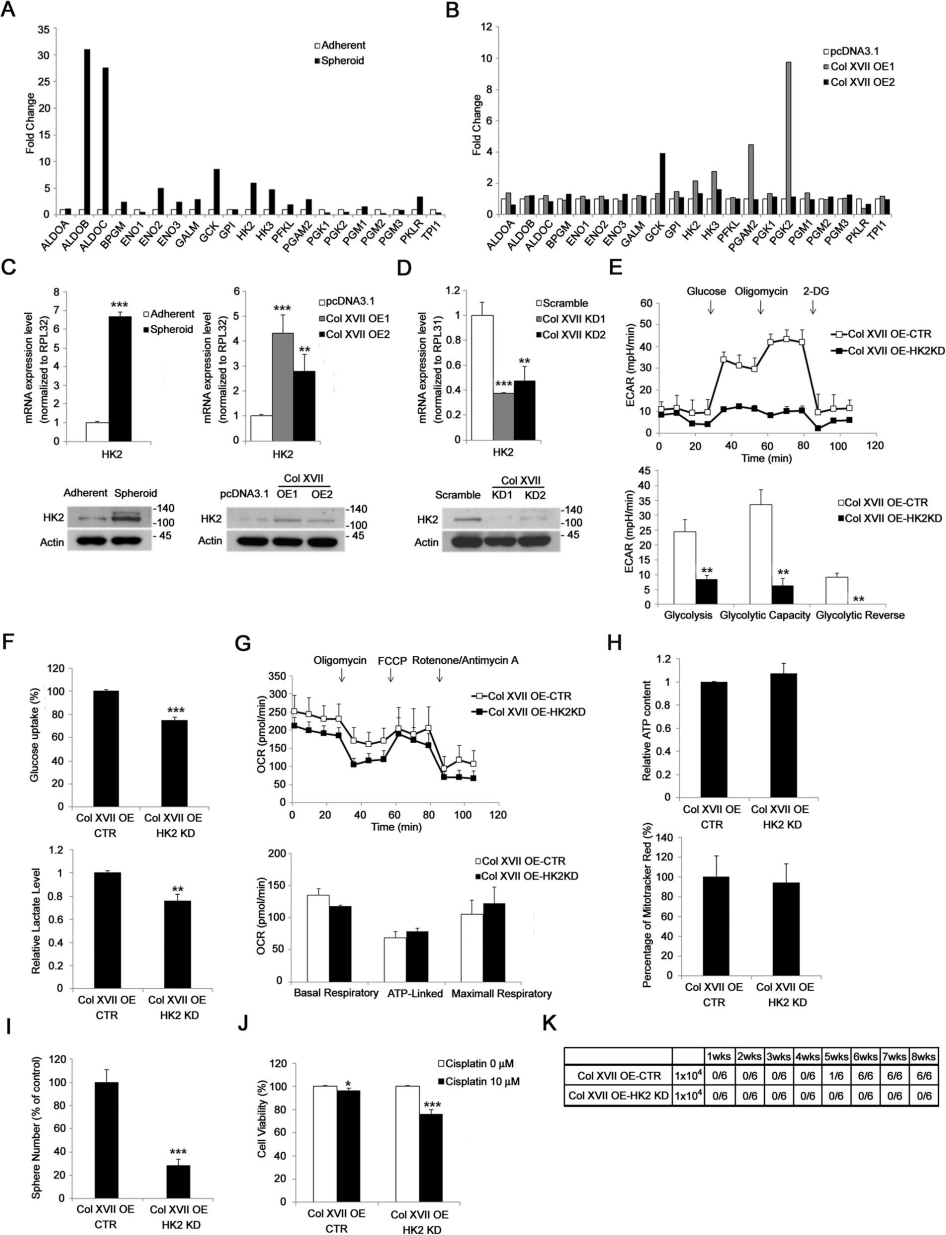
HK2 is required for collagen XVII-mediated increases of glycolysis and CSC features in lung cancer cells. **a** and **b,** PCR array showed increased expression of HK2 in both cells cultured in spheroid medium and cells with collagen XVII overexpression, indicating the HK2 plays an important role in collagen XVII-induced metabolic reprogramming in lung cancer cells. **c,** RT-PCR and western blot analysisshowed increased HK2 expression in both cells cultured in spheroid medium and cells with collagen XVII overexpression. **d,** RT-PCR and western blot analysis showed decreased expression of HK2 in cells with collagen XVII knockdown. **e,** Glycolysis was decreased in collagen XVII overexpressed lung cancer cells with HK2 knockdown. **f,** The glucose uptake and lactate level were decreased in collagen XVII overexpressed lung cancer cells with HK2 knockdown. **g,** However, OCR was not significantly different between collagen XVII overexpressed lung cancer cells with or without HK2 knockdown. **h,** The relative ATP content and the percentage of mitotracker red staining were neither different between collagen XVII overexpressed lung cancer cells with or without HK2 knockdown. **i,** The number of sphere formation decreased in collagen XVII overexpressed lung cancer cells with HK2 knockdown. **j,** Collagen XVII overexpressed lung cancer cells with HK2 knockdown were more sensitive to cisplatin. **k,** The capability of tumor initiation was decreased in collagen XVII overexpressed lung cancer cells with HK2 knockdown

To explore which factor mediated the role of collagen XVII in metabolic reprogramming of CSCs in lung cancer, we performed a quantitative RT-PCR array followed by confirmation with western blotting analysis and found that HK2 was upregulated in A549 spheroid culture as well as in cells with collagen XVII overexpression (Fig. 4a, b, and c). In contrast, HK2 expression in spheroid culture was downregulated when collagen XVII was silenced (Fig. 4d). HK2 silencing reduced ECAR (Fig. 4e), glucose uptake, and lactate production (Fig. 4f) in spheroid culture as well as adherent culture with collagen XVII overexpression. However, HK2 silencing did not reduce OCR (Fig. 4g), ATP production, and mitochondrial mass (Fig. 4h) in spheroid culture as well as adherent culture with collagen XVII overexpression. Moreover, HK2 silencing reduced the abilities of A549 with collagen XVII overexpression to form spheres in spheroid culture (Fig. 4i), resist chemotoxic agents (Fig. 4j), and form tumors in mouse xenograft models (Fig. 4k). These data suggest that HK2 is required for collagen XVII-mediated increase in glycolysis and the maintenance of CSC-like features in lung CSCs.

### Oct4 is required for collagen XVII-mediated increase of HK2 expression in lung CSCs

**Fig. 5:**
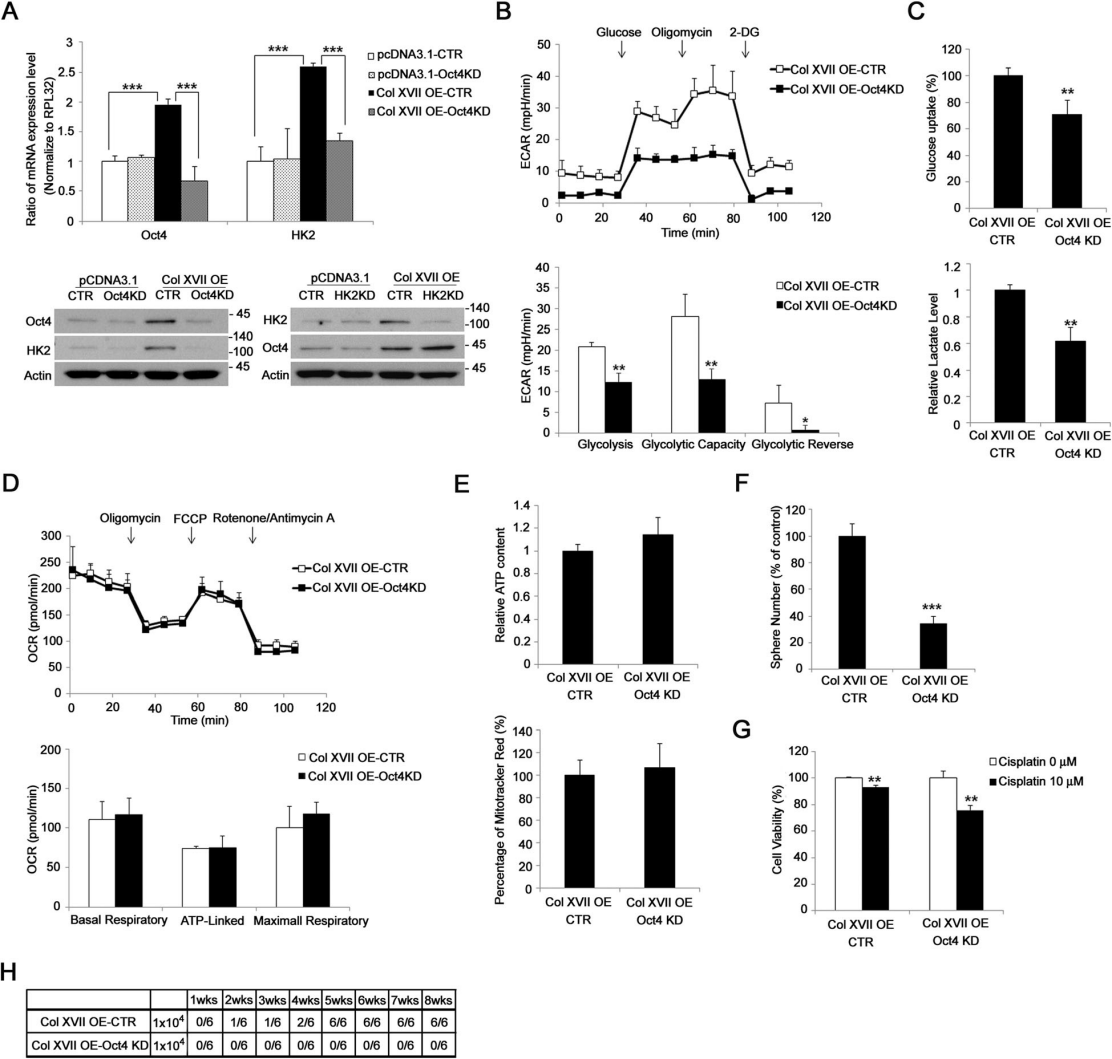
Oct4 acted as an upstream protein of HK2 and is also involved in the metabolic reprogramming of lung cancer cells with Collagen XVII overexpression. **a,** In lung cancer cells with collagen XVII overexpression, knockdown of Oct4 downregualted the mRNA and protein expression of HK2. **b,** Decreased glycosis in collagen XVII overexpressed lung cancer cells was noted when Oct4 was knockdown. **c,** The glucose uptake and lactate level were decreased in collagen overexpressed lung cancer cells with Oct4 knockdown. **d,** Oct4 did not affect the oxidative phosphorylation in lung cancer cells with collagen XVII overexpression. **e,** ATP content and the mitotracker red percentage were not different between collagen XVII overexpressed cells with or without Oct4 knockdown. **f,** Decreased sphere formation was observed in collagen XVII overexpressed lung cancer cells with Oct4 knockdown. **g,** Collagen XVII overexpressed cells with Oct4 knockdown were more sensitive to cisplatin treatment. **h**, The capability of tumor initiation was decreased in collagen XVII overexpressed lung cancer cells with Oct4 knockdown

To investigate the mechanism of collagen XVII-mediated increase in HK2 expression, we first demonstrated that spheroid culture or adherent culture with collagen XVII overexpression increased both the mRNA and the protein levels of Oct4 and HK2 (Fig. 5a, Additional file 4: Figure S4). Consistently, the expressions of Oct4 and HK2 were upregulated in xenograft tumors formed by A549 spheroid culture in comparion to adherent culture or by A549 transfected with collagen XVII vector compared to control vector (Additional file 5: Figure S5). Further, Oct4 silencing abrogated collagen XVII-mediated increase in HK2 expression (Fig. 5a). However, HK2 silencing did not block collagen XVII-mediated increase in Oct4 expression (Fig. 5a). Furthermore, Oct4 silencing blocked collagen XVII-mediated increase in ECAR (Fig. 5b), glucose uptake, and lactate production (Fig. 5c) compared to that in cells transfected with control shRNAs. Similar to HK2 silencing, Oct4 silencing did not block collagen XVII-mediated increase in OCR (Fig. 5d), ATP production, and mitochondrial mass (Fig. 5e) compared to that in cells transfected with control shRNAs. Also, Oct4 silencing reduced the abilities of A549 cells with collagen XVII overexpression to form spheres in spheroid culture (Fig. 5f), resist chemotoxic agents (Fig. 5g), and form tumors (Fig. 5h). These data together suggest that Oct4 is required for collagen XVII-mediated increase in HK2 expression, glycolysis, and the maintenance of CSC-like features in lung CSCs.

### Collagen XVII-laminin-332 pathway activates FAK-PI3K/AKT-GSK3β/β-catenin pathways to increase Oct4 expression

To investigate the mechanism underlying the collagen XVII-mediated increase in Oct4 expression, we first demonstrated that adherent culture with collagen XVII overexpression increased the phosphorylation levels of FAK, AKT and GSK3β (Additional file 6: Figure S6A). Further, treatment with FAK and PI3K inhibitor LY294002 blocked collagen XVII-mediated increase in Oct4 expression (Additional file 6: Figure S6B). Treatment with FAK inhibitor also reduced the phosphorylation levels of AKT and GSK3β (Additional file 6: Figure S6B). Treatment with LY294002 failed to reduce the phosphorylation level of FAK, while reduced the phosphorylation levels of AKT and GSK3β (Additional file 6: Figure S6B). Moreover, treatment with GSK3β inhibitor SB216763 increased β-catenin, Oct4, and HK2 levels, while treatment with selective Wnt/β-catenin signaling inhibitor ICG-001 blocked collagen XVII-mediated increase in β-catenin, Oct4, and HK2 expression (Additional file 6: Figure S6C). These data suggest that the collagen XVII-laminin-332 pathway activates the FAK-PI3K/AKT-GSK3β/β-catenin pathway to increase Oct4 expression, which subsequently increases HK2 expression and glycolysis.

### Collagen XVII-β-catenin-Oct4-HK2 pathway is also upregulated in CSCs of other lung cancer cells and other epithelial cancer type

To determine whether a similar pathway is also upregulated in CSCs of other lung cancer cells or in other epithelial cancers, we chose another human lung cancer cell line CL1–1 and a human colorectal cancer cell line HT-29 for further investigation. The spheroid culture of CL1–1 and HT-29 also showed an increase in the protein levels of collagen XVII, β-catenin, Oct4, and HK2 compared to the corresponding adherent culture (Additional file 7: Figure S7A). Interestingly, cells with collagen XVII overexpression also showed an increase in the protein levels of β-catenin, Oct4, and HK2 (Additional file 7: Figure S7B). Moreover, spheroid culture with collagen XVII silencing showed a reduction in the protein levels of β-catenin, Oct4, and HK2 in comparison with that observed with transfection with control shRNAs (Additional file 7: Figure S7C). These data suggest that the collagen XVII-β-catenin-Oct4-HK2 pathway is also upregulated in CSCs of other lung cancer cells and other epithelial cancer types.

### Expression of collagen XVII, Oct4, and HK2 predicts poorer prognosis in patients with lung cancer

**Fig. 6:**
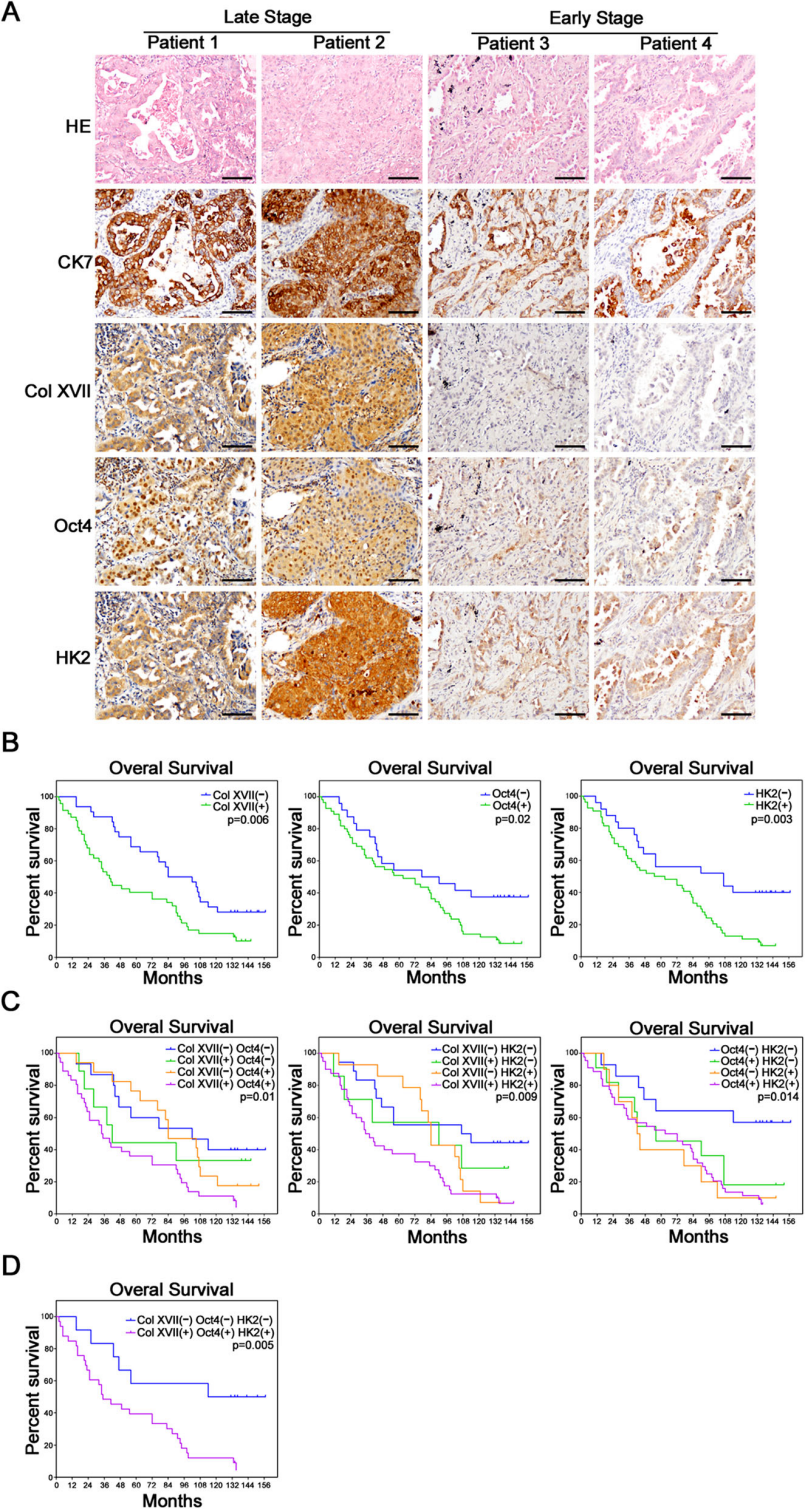
Collagen XVII, Oct4 and HK2 were associated with poor prognosis of lung cancer patients. **a,** The serial sections of immunohistochemical staining showed the patients with late stage had increased expression of Collagen XVII, Oct4 and HK2, and the patients with early stage had decreased expression of Collagen XVII, Oct4 and HK2. HE stain and CK7 immunostaining indicates tumor location. **b,** The survival curves of patients with lung cancer. The results showed patients with increased expressions of Collagen XVII, Oct4 and HK2 had worse prognosis than the patients with reduced expression of Collagen XVII, Oct4 and HK2. **c,** The survival curves of overall survival compared each group of patients with lung cancer cells, according to the expression of proteins. **d,** The patients with all increased expression of these 3 proteins had worst prognosis than the patients with all reduced expression of these 3 proteins. Scale bar, 50 μm

We used immunohistochemistry to investigate the clinical significance of collagen XVII, Oct4, and HK2 expression in tumor specimens from a cohort of 79 patients who received lung resection for lung cancer (Fig. 6a). The clinical demographics of the patients are described in Additional file 10: Table S2. Kaplan-Meier analysis showed that patients who displayed positive staining of either collagen XVII, Oct4 or HK2 (Fig. 6b) in their tumors exhibited poorer survival compared to patients who were negative for these markers. Patients who presented double-negative expression of collagen XVII and Oct4, or collagen XVII and HK2, or Oct4 and HK2 had a better prognosis than other groups (Fig. 6c). Patients who displayed triple-positive expression of collagen XVII, Oct4, and HK2 had a significantly worse prognosis than those with the triple-negative expression (Fig. 6d). These data suggest that collagen XVII, Oct4, and HK2 could be valuable markers to predict the prognosis of patients with lung cancer.

## Discussion

An outstanding issue in tumor cell biology is whether CSCs possess specific aberrant cellular characteristics, such as dysregulated metabolism, in a specific manner to maintain CSC-like features. For example, pancreatic CSCs have distinct metabolic phenotypes, relying on mitochondrial oxidative phosphorylation and show sensitivity to metformin-induced cell death, which are mechanistically determined by suppression of MYC and subsequent increase in peroxisome proliferator-activated receptor gamma coactivator 1 (PGC-1)α [22]. However, resistant CSC clones eventually emerged during metformin treatment and exhibited intermediate glycolytic/respiratory phenotype with balance between MYC and PGC-1α expressions. In this study, we demonstrated that collagen XVII mediates metabolic reprogramming in lung CSCs, increasing both glycolysis and oxidative phosphorylation. We then found that Oct4 and HK2 are upregulated in lung CSCs or lung cancer cells with collagen XVII overexpression. When Oct4 or HK2 was silenced, the metabolic alteration such as upregulated glycolysis was blocked and the CSC-like features were abrogated. These data confirmed that metabolic reprogramming is crucial for the maintenance of lung CSC-like features that depend on collagen XVII-mediated upregulation of Oct4 and HK2.

Collagen XVII is an essential component of the skin basement membrane. Mutation or autoantibodies targeting of collagen XVII leads to blistering skin disease [23]. In skin cancer, collagen XVII has been reported to be expressed in malignant but not in benign melanocytic tumor [24]. Increased collagen XVII expression is also associated with poorer outcome in colorectal cancer [25]. Aberrant *Col17A1* promoter methylation can predict the prognosis of patients with epithelial cancers, such as breast and cervical cancers [26]. Earlier reports from our group explain that collagen XVII is important to mediate suspension survival of CSCs and activate EMT-associated pathways [14, 15], both of which lead to tumor metastasis in lung cancer. In this study, we further demonstrated that collagen XVII is involved in metabolic reprogramming of CSCs in lung cancer and required for their survival and maintenance of CSC-like features. Taken together, collagen XVII has multiple roles in lung cancer progression and targeting collagen XVII and related signaling pathways could be a novel strategy to prevent metastasis in lung cancer patients. Moreover, the therapeutic agents should be delivered in a carrier, a micelle or a synthetic nanobody that is conjugated with an antibody against marker of CSC to prevent systemic destruction of hemidesmosomal adhesion, causing systemic skin blistering, and premature aging, since collagen XVII also plays an important role in normal stem cell niche functions [20, 21].

Recently, many studies have proposed that stem cells have unique metabolic profiles [27, 28]. Upregulation of glycolysis and downregulation of oxidative phosphorylation were first demonstrated in pluripotent stem cells [27]. Pluripotency factors, such as Oct4, directly upregulate HK2 and PKM2, which are important glycolytic enzymes that determine the glycolytic flux [11]. The overexpression of HK2 or PKM2 maintains high levels of glycolysis that hampers embryonic stem cell (ESC) differentiation and preserves the pluripotency of ESCs in the absence of leukemia inhibitory factor. Although it is clear that high glycolysis levels are important for pluripotency, their impact on CSC-like features, such as sphere formation in spheroid culture, chemoresistance, and tumorigenicity, remains unknown. We demonstrate that CSCs increase glycolysis by the Oct4-HK2 pathway which is activated by the FAK-PI3K/AKT-GSK3β/β-catenin signaling activated by the collagen XVII-laminin-332 pathway. We also demonstrate that CSCs are vulnerable to inhibitors of glycolysis, 2-DG, and galactose-induced apoptosis. Moreover, 2-DG decreased the abilities of CSCs to form spheres, resist chemotoxic agent-induced cell death, and form tumors in mouse xenograft models. These data suggest that CSCs, like ESCs, require upregulated glycolysis to maintain their stem cell-like features. Although PKM2 is upregulated by Oct4 in ESCs [11], its levels are not significantly upregulated in spheroid culture or adherent culture with collagen XVII overexpression (Additional file 8: Figure S8). These data suggest that pluripotent stem cells (PSCs) and CSCs share a similar mechanism to induce metabolic reprogramming and upregulate glycolysis. However, Oct4 downregulates oxidative phosphorylation in PSCs [11], while failing to downregulate or affect oxidative phosphorylation in CSCs, suggesting some dissimilarities between ESCs and CSCs.

## Conclusions

In conclusion, an aberrant upregulation of glycolysis and oxidative phosphorylation observed in lung CSCs is associated with the maintenance of CSC-like features. Activation of Oct4-HK2 pathway by collagen XVII-laminin-332 via activating FAK-PI3K/AKT-GSB3β/β-catenin is responsible for induced glycolysis and maintenance of CSC-like features in lung CSCs. More importantly, expression of collagen XVII, Oct4, and HK2 predicts a poorer prognosis in patients with lung cancer. Targeting the upregulated glycolysis, collagen XVII and its associated downstream pathways, and glycolytic genes could be novel strategies for eliminating CSCs and for enabling a better prognosis of cancer.

## Supplementary information

**Additional file 1: Fig. S1.** Collagen XVII plays an important role in the maintenance of CSC features in lung cancer cells. **A,** The picture of cell culture showed more spindle like shape cancer cells in of A549 lung cancer cells with collagen XVII cultured in adherent culture dishes and increased sphere formation in lung cancer cells with collagen XVII overexpression cultured in with spheroid medium (spheroid, CSC) for 12 days. After knock-out of collagen XVII in A549 cells, decreased sphere formation was noted in lung cancer cells with collagen XVII knockdown when cultured in spheroid culture. **B,** The results of flow cytometry showed increased ALDH activity in cells cultured in spheroid medium, in cells with collagen XVII overexpression in monolayer culture, and in cells with collagen XVII knockdown in spheroid culture. **C,** Western blot analysis of Oct4, Nanog and Sox2. **D,** Tumor initiation capabilities with TIC frequency of lung cancer cells in different culture system or cells with collagen XVII overexpression or knockdown. **E,** Increased lung metastasis when cells with collagen XVII overexpression injected from tail vein in animal models. **F,** Lung cancer cells with collagen XVII overexpression showed more chemoresistant, compared to cells without collagen XVII overexpression

**Additional file 2: Fig. S2.** Collagen XVII is essential for increased oxidative phosphorylation of lung cancer cells. **A,** Lung cancer cells A549 with collagen XVII overexpression showed increased oxygen consumption rate (OCR) compared to parental cells. **B** and **C,** The ATP content and mitotracker red staining showed increased ATP production and mitochondria mass in cells with collagen XVII overexpression. **D,** Decreased OCR was observed in lung cancer with collagen XVII knockdown. **E** and **F,** ATP content assay and Mitotracker Red staining showed decreased ATP production and mitochondria mass in cells with collagen XVII knockdown

**Additional file 3: Fig. S3.** Cell viability of lung cancer cells with galatose. **A,** Lung cancer cells cultured in spheroid medium were more resistant to galactose treatment. **B,** Two single clones of lung cancer cell with Collagen XVII overexpression were also more resistant to galactose treatment. **C,** Cells with collagen XVII knockdown in spheroid culture were more resistant to galactose treatment

**Additional file 4: Fig. S4.** Real time-PCR of glycolysis-related genes. Real time-PCR of glycolysis-related genes including HK2, HK3, GCK, PGAM2, and PGK2 in 4 single clones of lung cancer cells with collagen XVII overexpression

**Additional file 5: Fig. S5.** Additional file 1: H&E and IHC staining of xenograft tumor formed by A549 cells in adherent or spheroid culture, and A549 cells with collagen XVII overexpression or control pcDNA3.1 in adherent culture. CK7 immunostaining indicates tumor location. Scale bar, 50 μm

**Additional file 6: Fig. S6.** Collagen XVII activated FAK-AKT-GSK3β pathway, thus upregulated β-catenin and Oct4 in lung cancer cells with collagen XVII overexpression. **A,** Western blot analysis showed that increased FAK phosphrylation and the associated downstream proteins including AKT, GSK3β and β-catenin were all activated in collagen XVII overexpressed lung cancer cells. **B,** FAK inhibitor and PI3K inhibitor LY294002 were added in collagen XVII overexpressed cells to confirm Oct4 as the downstream of FAK-AKT pathway. **C,** Wnt/β-catenin inhibitor ICG-001 and GSK3 inhibitor SB216763 were added in collagen XVII overexpressed cells to confirm Oc4-HK2 as the downstream of GSK3β/β-catenin pathway

**Additional file 7: Fig. S7.** Western blot analysis of collagen XVII-β-catenin-Oct4-HK2 pathway in CL1–1 and HT-29 cells. **A,** Western blot analysis of collagen XVII-β-catenin-Oct4-HK2 pathway in CL1–1 and HT-29 cells in spheroid culture. **B,** Western blot analysis of collagen XVII-β-catenin-Oct4-HK2 pathway in CL1–1 and HT-29 cells with collagen XVII overexpression in monolayer culture. **C,** Western blot analysis of collagen XVII-β-catenin-Oct4-HK2 pathway in CL1–1 and HT-29 cells with collagen XVII knockdown in spheroid culture

**Additional file 8: Fig. S8.** Western blot analysis of PKM2 of cells in different culture systems and cells with collagen XVII overexpression or knockdown

**Additional file 9: Table S1.** Primer sequence for RT-PCR

**Additional file 10: Table S2.** Demographic data of 79 patients who underwent surgery for lung cancer

## Data Availability

Data and materials related to this study are available from the corresponding author on reasonable request.
