# Supplementary figures and images for: Involvement of collagen XVII in pluripotency gene expression and metabolic reprogramming of lung cancer stem cells
Source: J Biomed Sci. 2020 Jan 13;27:5. doi: 10.1186/s12929-019-0593-y (PMC6956558; doi:10.1186/s12929-019-0593-y)

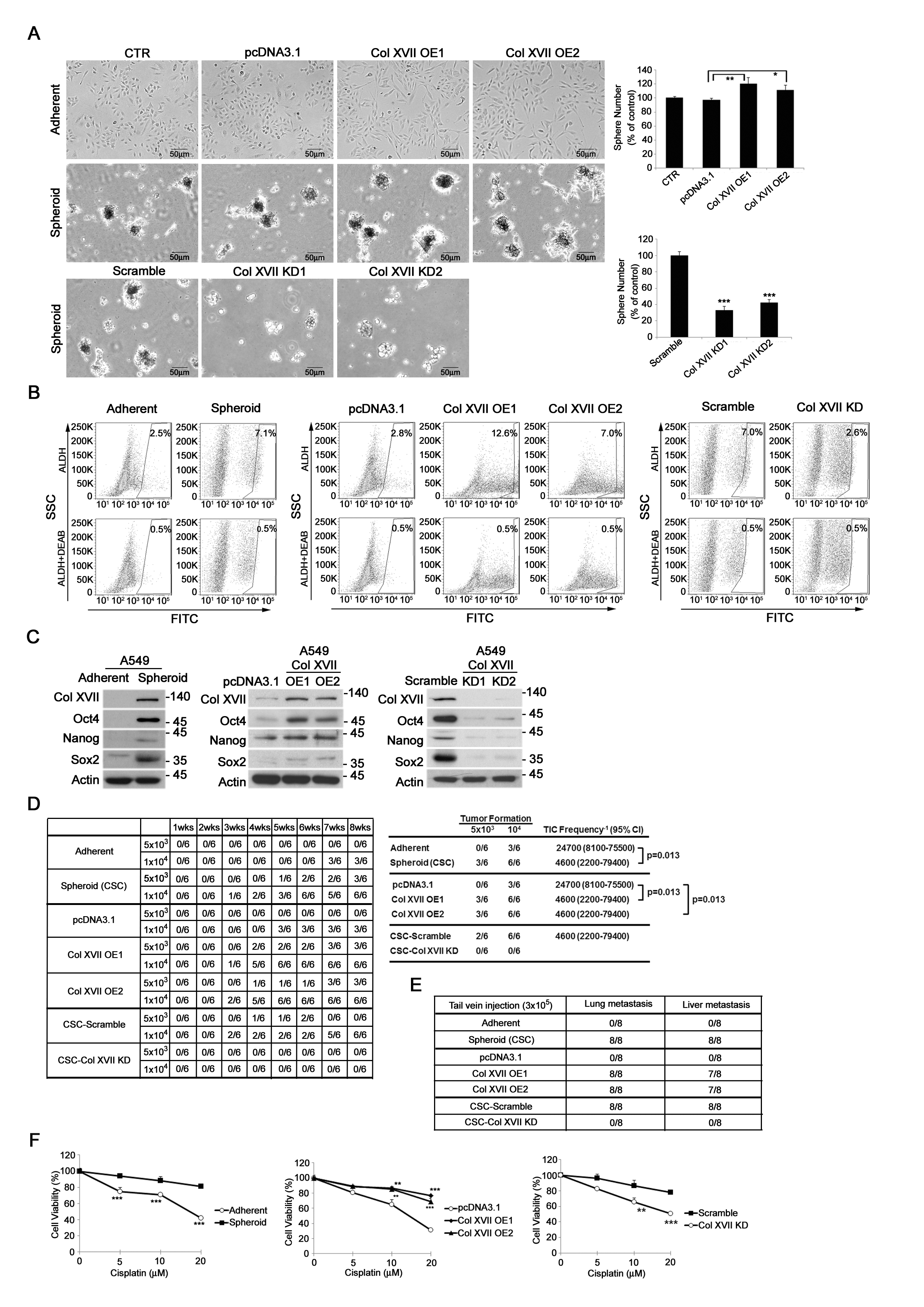

Supplement: Supplementary file 1 — Additional file 1: Fig. S1. Collagen XVII plays an important role in the maintenance of CSC features in lung cancer cells. A, The picture of cell culture showed more spindle like shape cancer cells in of A549 lung cancer cells with collagen XVII cultured in adherent culture dishes and increased sphere formation in lung cancer cells with collagen XVII overexpression cultured in with spheroid medium (spheroid, CSC) for 12 days. After knock-out of collagen XVII in A549 cells, decreased sphere formation was noted in lung cancer cells with collagen XVII knockdown when cultured in spheroid culture. B, The results of flow cytometry showed increased ALDH activity in cells cultured in spheroid medium, in cells with collagen XVII overexpression in monolayer culture, and in cells with collagen XVII knockdown in spheroid culture. C, Western blot analysis of Oct4, Nanog and Sox2. D, Tumor initiation capabilities with TIC frequency of lung cancer cells in different culture system or cells with collagen XVII overexpression or knockdown. E, Increased lung metastasis when cells with collagen XVII overexpression injected from tail vein in animal models. F, Lung cancer cells with collagen XVII overexpression showed more chemoresistant, compared to cells without collagen XVII overexpression [file 12929_2019_593_MOESM1_ESM.tif]

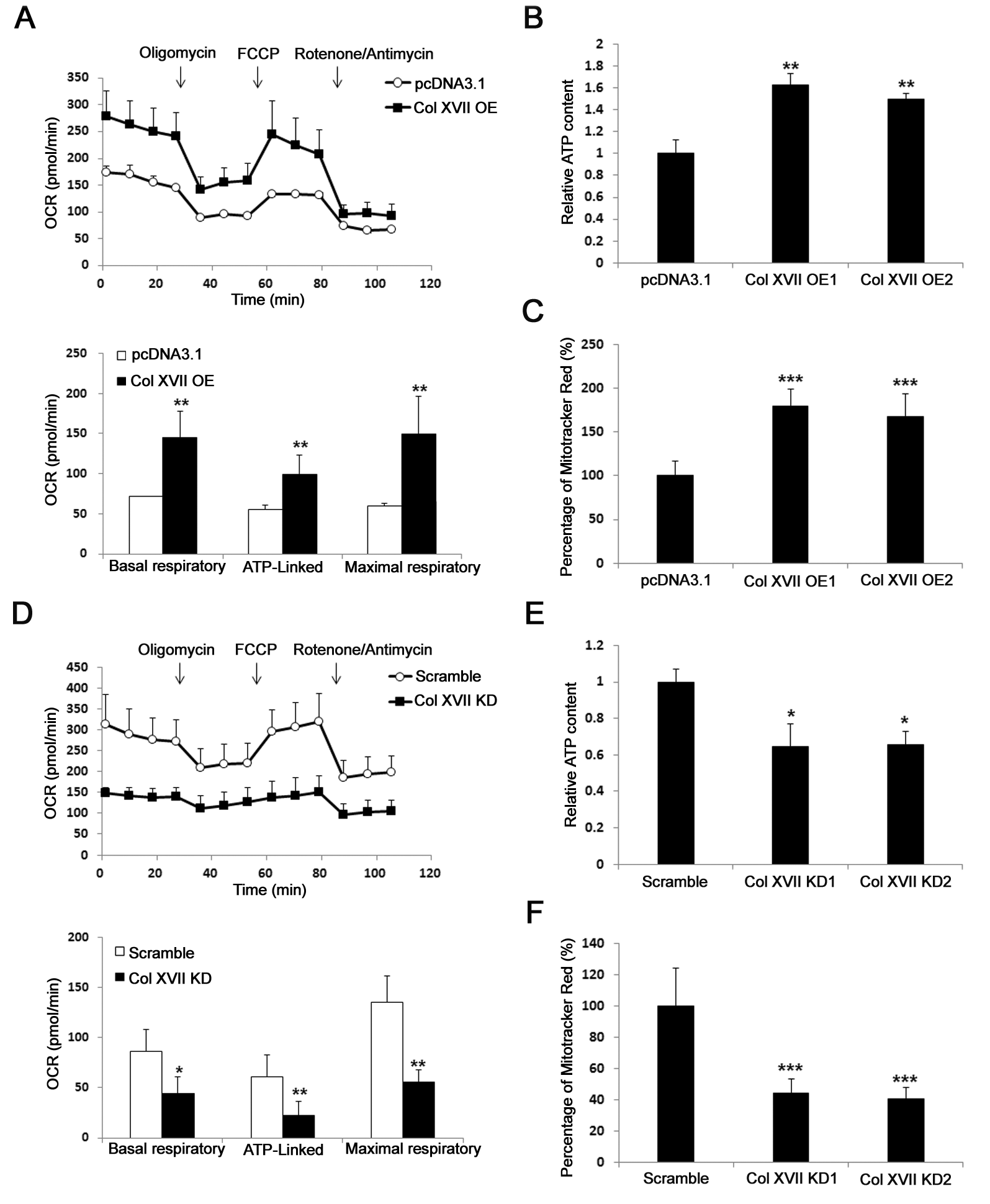

Supplement: Supplementary file 2 — Additional file 2: Fig. S2. Collagen XVII is essential for increased oxidative phosphorylation of lung cancer cells. A, Lung cancer cells A549 with collagen XVII overexpression showed increased oxygen consumption rate (OCR) compared to parental cells. B and C, The ATP content and mitotracker red staining showed increased ATP production and mitochondria mass in cells with collagen XVII overexpression. D, Decreased OCR was observed in lung cancer with collagen XVII knockdown. E and F, ATP content assay and Mitotracker Red staining showed decreased ATP production and mitochondria mass in cells with collagen XVII knockdown [file 12929_2019_593_MOESM2_ESM.tif]

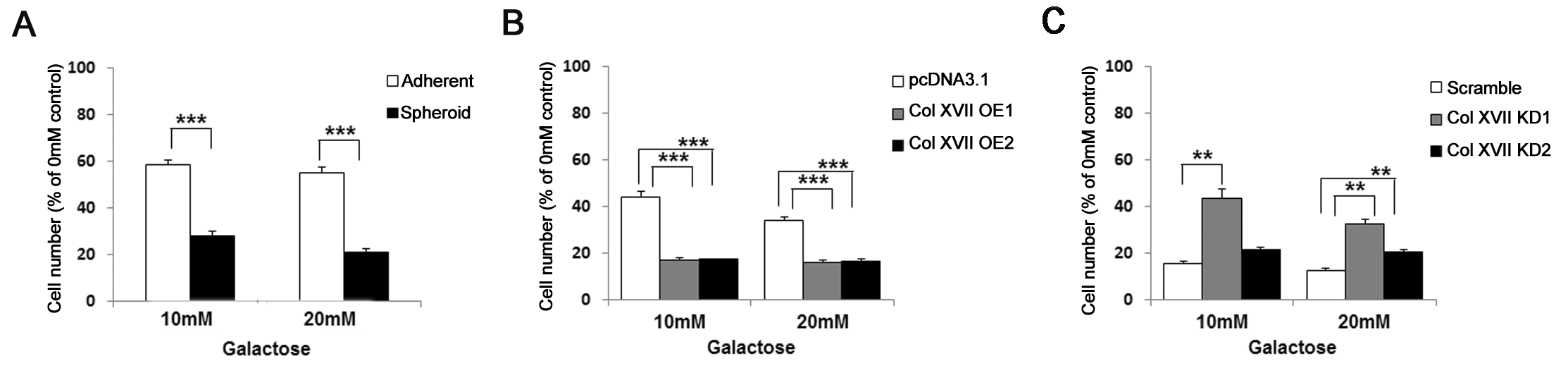

Supplement: Supplementary file 3 — Additional file 3: Fig. S3. Cell viability of lung cancer cells with galatose. A, Lung cancer cells cultured in spheroid medium were more resistant to galactose treatment. B, Two single clones of lung cancer cell with Collagen XVII overexpression were also more resistant to galactose treatment. C, Cells with collagen XVII knockdown in spheroid culture were more resistant to galactose treatment [file 12929_2019_593_MOESM3_ESM.tif]

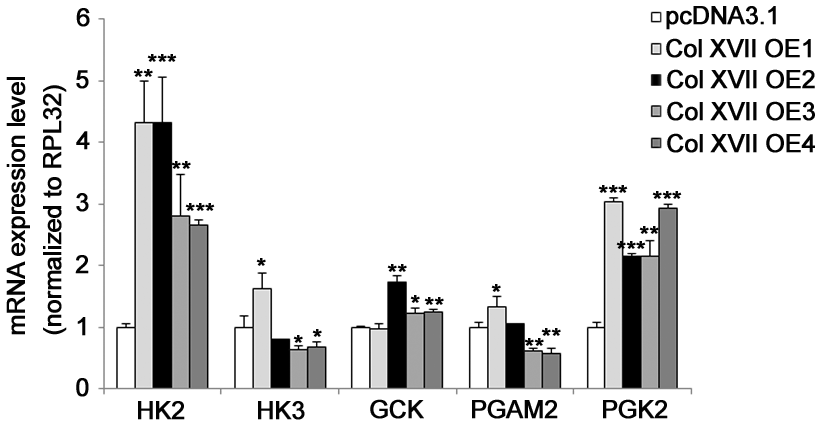

Supplement: Supplementary file 4 — Additional file 4: Fig. S4. Real time-PCR of glycolysis-related genes. Real time-PCR of glycolysis-related genes including HK2, HK3, GCK, PGAM2, and PGK2 in 4 single clones of lung cancer cells with collagen XVII overexpression [file 12929_2019_593_MOESM4_ESM.tif]

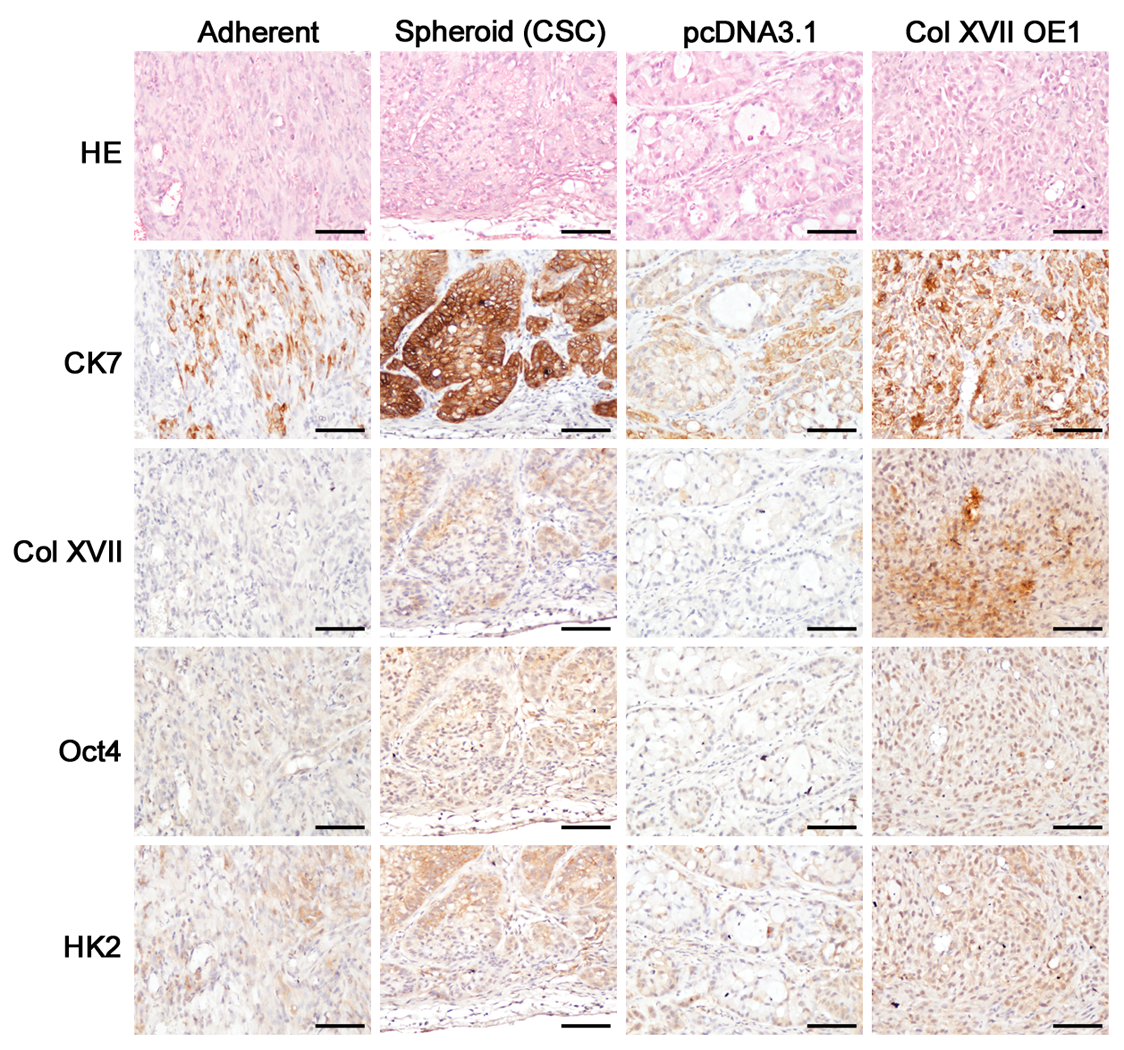

Supplement: Supplementary file 5 — Additional file 5: Fig. S5. Additional file 1: H&E and IHC staining of xenograft tumor formed by A549 cells in adherent or spheroid culture, and A549 cells with collagen XVII overexpression or control pcDNA3.1 in adherent culture. CK7 immunostaining indicates tumor location. Scale bar, 50 μm [file 12929_2019_593_MOESM5_ESM.tif]

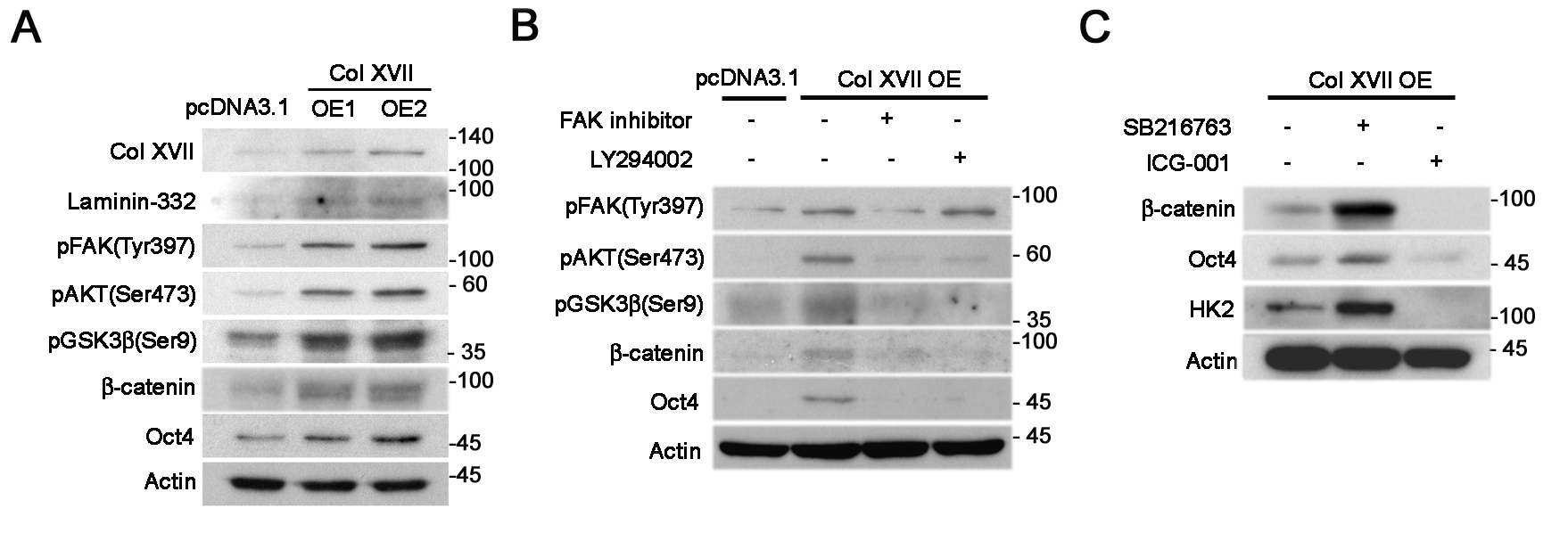

Supplement: Supplementary file 6 — Additional file 6: Fig. S6. Collagen XVII activated FAK-AKT-GSK3β pathway, thus upregulated β-catenin and Oct4 in lung cancer cells with collagen XVII overexpression. A, Western blot analysis showed that increased FAK phosphrylation and the associated downstream proteins including AKT, GSK3β and β-catenin were all activated in collagen XVII overexpressed lung cancer cells. B, FAK inhibitor and PI3K inhibitor LY294002 were added in collagen XVII overexpressed cells to confirm Oct4 as the downstream of FAK-AKT pathway. C, Wnt/β-catenin inhibitor ICG-001 and GSK3 inhibitor SB216763 were added in collagen XVII overexpressed cells to confirm Oc4-HK2 as the downstream of GSK3β/β-catenin pathway [file 12929_2019_593_MOESM6_ESM.tif]

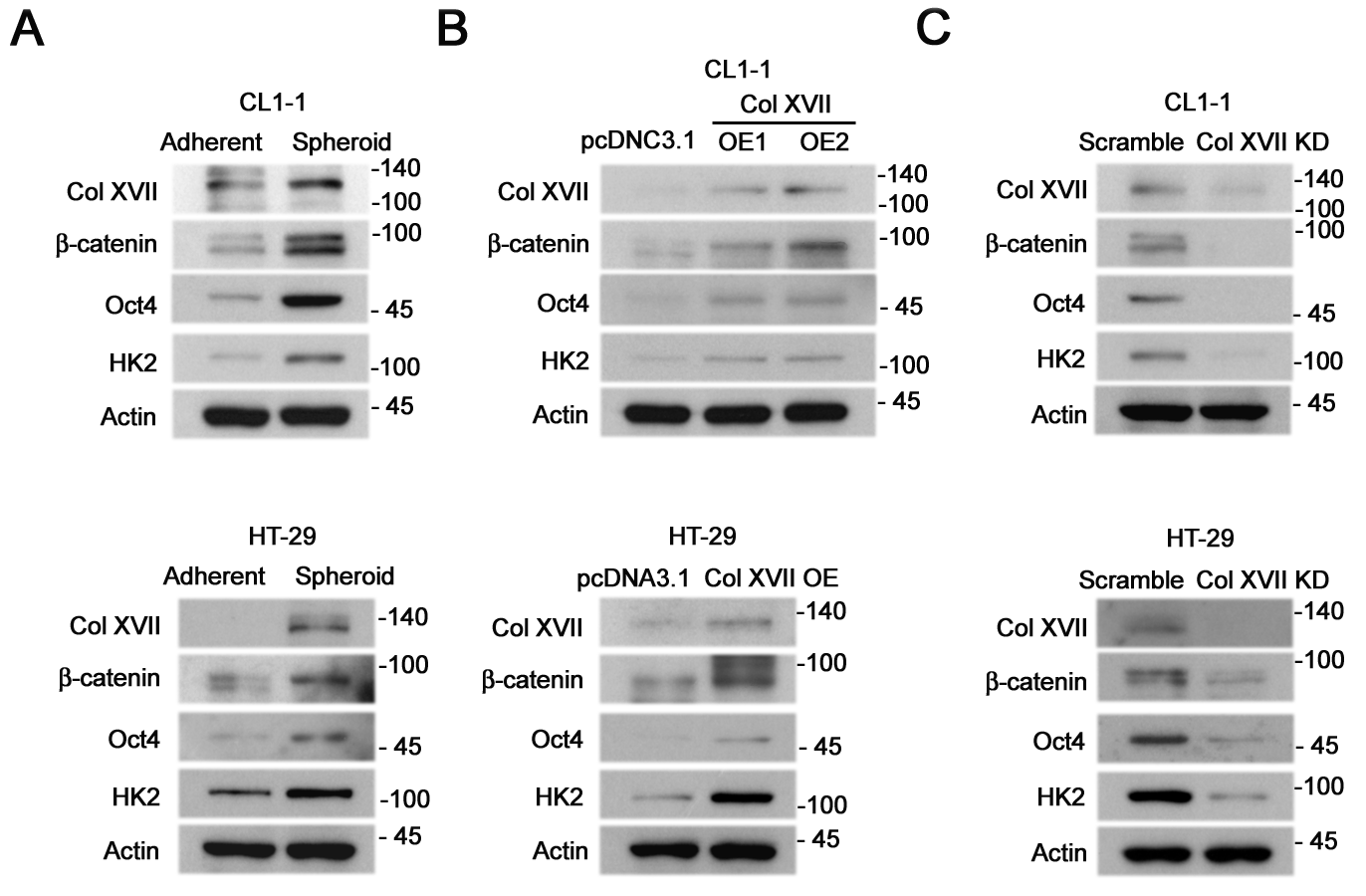

Supplement: Supplementary file 7 — Additional file 7: Fig. S7. Western blot analysis of collagen XVII-β-catenin-Oct4-HK2 pathway in CL1–1 and HT-29 cells. A, Western blot analysis of collagen XVII-β-catenin-Oct4-HK2 pathway in CL1–1 and HT-29 cells in spheroid culture. B, Western blot analysis of collagen XVII-β-catenin-Oct4-HK2 pathway in CL1–1 and HT-29 cells with collagen XVII overexpression in monolayer culture. C, Western blot analysis of collagen XVII-β-catenin-Oct4-HK2 pathway in CL1–1 and HT-29 cells with collagen XVII knockdown in spheroid culture [file 12929_2019_593_MOESM7_ESM.tif]

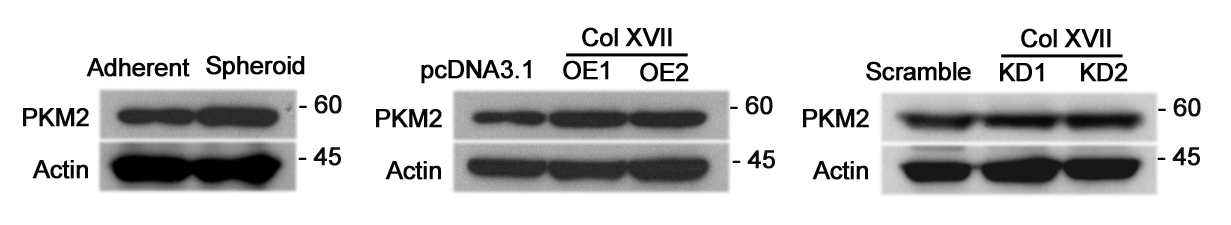

Supplement: Supplementary file 8 — Additional file 8: Fig. S8. Western blot analysis of PKM2 of cells in different culture systems and cells with collagen XVII overexpression or knockdown [file 12929_2019_593_MOESM8_ESM.tif]
