# Supplementary material for: Involvement of collagen XVII in pluripotency gene expression and metabolic reprogramming of lung cancer stem cells
Source: J Biomed Sci. 2020 Jan 13;27:5. doi: 10.1186/s12929-019-0593-y (PMC6956558; doi:10.1186/s12929-019-0593-y)
Supplement: Supplementary file 9 — Additional file 9: Table S1. Primer sequence for RT-PCR [file 12929_2019_593_MOESM9_ESM.docx]

**Supplementary Table 1. Primer sequence for RT-PCR**

| **Primer name** | **Primer sequences** |
| --- | --- |
| **GCK** | **F: 5'-CATCTCCGACTTCCTGGACAAG-3'**  **R: 5'-TGGTCCAGTTGAGAAGGATGCC -3'** |
| **HK2** | **F: 5'- TGGAGATGGAGAATCAGA-3'**  **R: 5'- CCAGGAAACTCTCGTCTA-3'** |
| **HK3** | **F: 5'-** **TTTGACTTTGCTGCCCACTG -3'**  **R: 5'-** **AAGAGAAGCTGAAGCCAAGC -3'** |
| **Oct4** | **F: 5'-** **CGCAAGCCCTCATTTCAC -3'**  **R: 5'- CATCACCTCCACCACCTG-3'** |
| **PGAM2** | **F: 5'- CCCTTCTGGAACGAGGAGATT-3'**  **R: 5'-AGGTTCAGCTCCATGATCGCCT -3'** |
| **PGK2** | **F: 5'-TGACGAGAACGCTCAGGTTGGA -3'**  **R: 5'- GCCTTGCTTGAGCCACAACTTG-3'** |
| **RPL32** | **F: 5'-** **CATCTCCTTCTCGGCATCA -3'**  **R: 5'- AACCCTGTTGTCAATGCCTC-3'** |
