# Supplementary material for: Involvement of collagen XVII in pluripotency gene expression and metabolic reprogramming of lung cancer stem cells
Source: J Biomed Sci. 2020 Jan 13;27:5. doi: 10.1186/s12929-019-0593-y (PMC6956558; doi:10.1186/s12929-019-0593-y)
Supplement: Supplementary file 10 — Additional file 10: Table S2. Demographic data of 79 patients who underwent surgery for lung cancer [file 12929_2019_593_MOESM10_ESM.docx]

| Table1 | **Supplementary table 2. Demographic data of 79 patients who underwent surgery**  **for lung cancer** | | | | | | |  |
| --- | --- | --- | --- | --- | --- | --- | --- | --- |
| Variables (*n*=79) | | |  |  |  | Number (%) | |  |
| Age (year) | |  |  |  |  | 41-92 (66.5 ± 11.2) | |  |
| Sex | |  |  |  |  |  |  |  |
|  | Male |  |  |  |  | 54 (68.4) |  |  |
|  | Female |  |  |  |  | 25 (31.6) |  |  |
| Histological type | | |  |  |  |  |  |  |
|  | Adenocarcinoma | |  |  |  | 52 (65.8) |  |  |
|  | Squamous cell carcinoma | | |  |  | 18 (22.8) |  |  |
|  | Large cell carcinoma | |  |  |  | 3 (3.8) |  |  |
|  | Mucoepidermoid carcinoma | | |  |  | 3 (3.8) |  |  |
|  | Others |  |  |  |  | 3 (3.8) |  |  |
| T status | |  |  |  |  |  |  |  |
|  | T1 |  |  |  |  | 14 (17.7) |  |  |
|  | T2 |  |  |  |  | 58 (73.4) |  |  |
|  | T3 |  |  |  |  | 1 (1.3) |  |  |
|  | T4 |  |  |  |  | 6 (7.6) |  |  |
| N status | |  |  |  |  |  |  |  |
|  | N0 |  |  |  |  | 45 (57) |  |  |
|  | N1 |  |  |  |  | 10 (12.7) |  |  |
|  | N2 |  |  |  |  | 24 (30.4) |  |  |
| M status | |  |  |  |  |  |  |  |
|  | M0 |  |  |  |  | 76 (96.2) |  |  |
|  | M1 |  |  |  |  | 3 (3.8) |  |  |
| Collagen XVII expression | | |  |  |  |  |  |  |
|  | Increased expression (+) | | |  |  | 47 (59.5) |  |  |
|  | Decreased expression (-) | | |  |  | 32 (40.5) |  |  |
| Oct 4 expression | | |  |  |  |  |  |  |
|  | Increased expression (+) | | |  |  | 55 (69.6) |  |  |
|  | Decreased expression (-) | | |  |  | 24 (30.4) |  |  |
| HK 2 expression | | |  |  |  |  |  |  |
|  | Increased expression (+) | | |  |  | 54 (68.4) |  |  |
|  | Decreased expression (-) | | |  |  | 25 (31.6) |  |  |
| Collagen XVII, Oct 4 and HK 2 coexpression | | | | |  |  |  |  |
|  | Collagen XVII (+)/Oct 4 (+)/HK 2 (+) | | | |  | 33 (41.8) |  |  |
|  | Collagen XVII (+)/Oct 4 (+)/HK 2 (-) | | | |  | 5 (6.3) |  |  |
|  | Collagen XVII (+)/Oct 4 (-)/HK 2 (+) | | | |  | 7 (8.9) |  |  |
|  | Collagen XVII (+)/Oct 4 (-)/HK 2 (-) | | | |  | 2 (2.5) |  |  |
|  | Collagen XVII (-)/Oct 4 (+)/HK 2 (+) | | | |  | 11 (13.9) |  |  |
|  | Collagen XVII (-)/Oct 4 (+)/HK 2 (-) | | | |  | 6 (7.6) |  |  |
|  | Collagen XVII (-)/Oct 4 (-)/HK 2 (+) | | | |  | 3 (3.8) |  |  |
|  | Collagen XVII (-)/Oct 4 (-)/HK 2 (-) | | | |  | 12 (15.2) |  |  |
